# Mouse Model of Anti-Obesity Effects of *Blautia hansenii* on Diet-Induced Obesity

**DOI:** 10.3390/cimb45090452

**Published:** 2023-08-26

**Authors:** Masaki Shibata, Naoki Ozato, Harutoshi Tsuda, Kenta Mori, Keita Kinoshita, Mitsuhiro Katashima, Yoshihisa Katsuragi, Shigeyuki Nakaji, Hayato Maeda

**Affiliations:** 1 Faculty of Agriculture and Life Science, Hirosaki University, 3 Bunkyo-cho, Hirosaki 036-8561, Japan; iu3222004@hirosaki-u.ac.jp (M.S.); tsudah@hirosaki-u.ac.jp (H.T.); 2 The United Graduate School of Agricultural Sciences, Iwate University, 3-18 Ueda, Morioka 020-0066, Japan; 3 Health & Wellness Products Research Laboratories, Kao Corp., 2-1-3 Bunka, Sumida-ku 131-8501, Japan; oozato.naoki@kao.com (N.O.); mori.kenta@kao.com (K.M.); kinoshita.keita@kao.com (K.K.); katashima.mitsuhiro@kao.com (M.K.); katsuragi.yoshihisa@kao.com (Y.K.); 4 Department of Social Medicine, Graduate School of Medicine, Hirosaki University, 5 Zaifu-cho, Hirosaki 036-8562, Japan; nakaji@hirosaki-u.ac.jp; 5 Institute of Regional Innovation, Hirosaki University, 2-1-1 Yanagawa, Aomori 038-0012, Japan

**Keywords:** *Blautia hansenii*, insulin resistance, intestinal microflora, obesity, visceral fat

## Abstract

Reportedly, a relationship exists between intestinal microflora and obesity-related lifestyle diseases. *Blautia* spp. a major intestinal microbiota, accounts for 3–11% of human intestinal microflora. Epidemiological reports have described that people with more visceral fat have less *Blautia hansenii* in their intestinal tract irrespective of age or gender. However, the effect of oral administration of heat-sterilized *Blautia hansenii* on obesity has not been clarified. Therefore, the aim of this study was to evaluate the effects of dietary *Blautia hansenii* administration on obesity in high-fat-diet-induced obesity in a mouse model. Heat-sterilized cells of *Blautia hansenii* were used. C57BL/6J mice (normal mice, *n* = 7) were fed with each experimental diet for nine weeks. Diets for experimentation were: normal-fat (NF) diets, high-fat (HF) diets, and high-fat + *Blautia hansenii* (HF + *Blautia*) diets. The HF + *Blautia* group was administered about 1 × 10^9^ (CFU/mouse/day) of *Blautia hansenii.* During the periods of experimentation, body weight, food intake, water consumption, and fecal weight were recorded, and glucose tolerance tests were performed. Subsequently, the white adipose tissue (WAT) weight and serum components were measured. Short-chain fatty acid contents in the feces and cecum were analyzed. Furthermore, changes in the intestinal microflora were analyzed using meta-genomics analysis. Results showed that the total weight of WAT in the HF + *Blautia* group was significantly lower (13.2%) than that of the HF group. Moreover, the HF + *Blautia* group exhibited better glucose tolerance than the HF group. Productivity of short-chain fatty acids in the intestinal tract was at a significantly (*p* < 0.05) low level in the HF group; on the other hand, it recovered in the HF + *Blautia* group. Furthermore, there was a higher ratio of *Blautia* (*p* < 0.05) in the intestinal tracts of the HF + *Blautia* group than in the HF group. These results suggest that *Blautia hansenii* administration suppresses obesity induced by a high-fat diet.

## 1. Introduction

Obesity is major risk factor leading to metabolic syndrome and related disorders [1,2]. In particular, adipocytokines secreted by obesity-condition adipocyte cells, induce insulin resistance, hypertension, and dyslipidemia [3]. Therefore, it is important to reduce the amounts of visceral fat to prevent obesity-related diseases. Various obesity-prevention studies are underway, one of which is regulation of intestinal bacteria.

Estimates indicate that approximately 1000 bacterial species exist along with archaea, fungi, and viruses in the human intestinal tract [4]. Human intestinal microflora comprise more than 100 trillion bacteria [5]. The gut microbiota have been revealed as a complex environment that maintains a delicate balance similar to that of microorganisms in an ecosystem. In humans, four major phyla exist: Firmicutes, Bacteroidetes, Actinobacteria, and Proteobacteria [6].

A relationship between intestinal microflora and several diseases has been clarified [5,7]. In recent years, modern sequencing technology has facilitated detailed analyses of intestinal flora. Several reports have described that intestinal microflora affect colorectal cancer, neurological disorders such as multiple sclerosis, and autism-spectrum disorders [5]. Furthermore, microflora are associated with host energy regulation and homeostasis. These microflora compositions are affected by environmental factors such as age, gender, and dietary habits [8,9,10]. In particular, intestinal microbiota metabolites from dietary fiber show beneficial effects for host metabolism and homeostasis [11]. These microbiota secrete large amounts of metabolizing compounds such as acetic acid, butyric acid, propionic acid, and lactic acid. These short-chain fatty acids maintain acidic conditions in the intestinal tract and suppress the growth of harmful intestine microbiota. After these short-chain fatty acids are absorbed into the body in the large intestine, they become involved in regulating appetite, lipid metabolism, and glucose homeostasis [11,12]. For example, consumption of probiotics such as lactic acid bacteria has preventive and ameliorating effects on several diseases [13,14,15]. Further, *Lactobacillus* and *Bifidobacterium* consumption prevent body-fat accumulation [16,17,18].

Several reports have described that intestinal microflora of healthy and obese individuals differ. The most well-known finding is that obese people show higher Firmicutes/Bacteroidetes ratios than those of normal-weight individuals [19]. However, this finding is apparently limited to women. Some reports have presented opposite results obtained from animal and epidemiological studies [20,21,22,23]. Therefore, studies of the relationship between obesity and intestinal microflora at the phylum level have produced unclear findings.

Ozato et al. reported that *Blautia hansenii* in intestinal microflora are significantly correlated with visceral fat area (VFA) and body mass index (BMI), irrespective of gender or age. This study, a longitudinal study of 767 people in Japan [24,25], found *Blautia hansenii* to be negatively associated with VFA accumulation. Other reports suggest that *Blautia* in intestinal microbiota affects metabolic diseases, inflammatory diseases, and biotransformation [26].

Members of *Blautia* spp., an abundant genus in the gut, comprise about 3–11% of human intestinal microbiota, irrespective of race [24,27,28]. Reportedly, the relative frequency of *Blautia* in gut microflora is lower in patients with diabetes, liver cirrhosis, colorectal cancer, and rheumatoid arthritis [24,25]. *Blautia* bacteria produce butyric acid and acetic acid, promoting metabolism and stimulating G-protein-coupled receptors (GPR) 41 and 43 [29]. GPR is a short-chain fatty acid receptor. GPR 41 is expressed in sympathetic nerves; its ligand compounds promote energy expenditure, with accompanying increased heart rate and heat production [30]. GPR 43 is expressed in white adipose tissue. Its stimulation induces suppression of the absorption of glucose and fatty acids in adipocytes, thereby regulating insulin sensitivity [31].

The abundance of *Blautia*, which is less abundant in the intestinal microflora of infants, increases with age [32]. Therefore, it is presumably taken into gut microbiota from food and becomes established thereafter. The abundance of *Blautia* in the gut microbiota is reportedly higher in Asian people than in Western people. Moreover, some reports describe that it increases with the intake of traditional Asian foods such as *Koji* mold and natto (fermented soybeans). Therefore, *Blautia* are considered to migrate from these traditional fermented foods. Dietary prebiotics such as fructooligosaccharides reportedly promote the growth of *Blautia coccoides* in the intestinal tract [33]. Additionally, it has been reported recently that oral administration of *Blautia wexlerae* has a preventive effect against obesity and type 2 diabetes in mice [34]. Nevertheless, no reports have described an evaluation of functionality by oral administration of *Blautia hansenii*, which has been correlated with visceral fat and blood sugar concentrations based on results of epidemiological studies. Furthermore, no reports have described the functionality of heat-sterilized bacteria by oral administration. Several lactic acid bacteria reportedly improved intestinal inflammation caused by heat-sterilized bacteria [35]. Actually, heat-sterilized bacteria are easy to handle and transport, and are useful as food ingredients. If the functionality of heat-sterilized bacteria were demonstrated, then it might be possible to use the bacteria as a functional food material.

The aim of this study was to analyze the effects of dietary ingestion of heat-sterilized *Blautia* on a mouse model of diet-induced obesity. After C57BL/6J mice had consumed high-fat diets containing heat-sterilized *Blautia* for nine weeks, their white adipose tissue (WAT) weights and their glucose tolerance levels were examined. Furthermore, the concentrations of short-chain fatty acids in the feces and cecum were analyzed. Additionally, intestinal microflora of feces were analyzed using metagenomics analysis.

## 2. Materials and Methods

### 2.1. Samples and Reagents

*Blautia hansenii* (JCM00014655) were obtained from the Riken Bio Resource Research Center (Ibaraki, Japan). The bacteria were cultured in GAM Broth (Nippon Suisan Kaisha, Ltd., Tokyo, Japan), modified with 5% CO_2_ at 37 °C. After 48 h, the cultured medium was centrifuged at 500 rpm. Then, the precipitate was collected and washed with physiological saline. Subsequently, it was sterilized at 121 °C for 15 min using an autoclave. Then, sterilized bacteria were collected again by centrifugation. The precipitate was freeze-dried using a freeze dryer and used for addition to animal diets.

All chemicals, guaranteed to be of reagent or tissue-culture grade, were from Sigma (St. Louis, MO, USA) or from Wako Pure Chemical Industries Ltd. (Osaka, Japan).

### 2.2. Animal Diet and Care

C57BL/6J mice (male, 4 weeks old) were purchased from CLEA Japan, Inc. (Tokyo, Japan). The animals were housed in an air-controlled room (temperature, 23 ± 1 °C) with a 12/12 h light/dark cycle. Mice were provided with ad libitum access to standard diet and tap water. After adaptation for 1 week, mice (*n* = 7 mice/group) were fed with the experimentation diet for 9 weeks. The experimentation diet was prepared according to the recommendations of AIN-93G. The diet compositions are presented in Table 1. The nutritional value of the NF group’s diet was 2518 kcal/kg of carbohydrate, 800 kcal/kg of protein, 630 kcal/kg of fat, and total calories were 3948 kcal/kg. The nutritional value of the HF group’s and HF + *Blautia* group’s diet was 1878 kcal/kg of carbohydrate, 920 kcal/kg of protein, 1800 kcal/kg of fat, and total calories were 4598 kcal/kg. Subsequently, mice were killed after being anesthetized with isoflurane. Blood was collected from the abdominal aorta and serum was prepared for biochemical analysis. Liver, kidney, spleen, brown adipose tissue (BAT), and epididymal, mesenteric, inguinal, perirenal, and retroperitoneal white adipose tissue (WAT) were removed rapidly, weighed, and frozen in liquid nitrogen, followed by storage at −70 °C until analysis. For analysis of mRNA expression, some epididymal WAT was stored in RNAlater™ solution (Thermo Fisher Scientific K.K., Tokyo, Japan) at −70 °C. Serum components (GOT, GPT, and triglyceride) were analyzed using an automatic analyzer (Olympus AU5431; Olympus Corp., Tokyo, Japan) at the Japan Medical Laboratory (Osaka, Japan). The blood glucose concentration was analyzed using NIPRO Stat Strip XP2 (Nipro Corp., Osaka, Japan). Serum peptide YY concentrations were analyzed with a Mouse/Rat PYY ELISA kit Wako (Fujifilm Wako Pure Chemical Corp., Osaka, Japan) according to the manufacturer’s instructions.

### 2.3. Glucose Tolerance Tests

Glucose tolerance tests were conducted after 9 weeks of the experimental period. Mice were starved for 7 h, followed by ingestion of 1.5 g/kg body weight of glucose. After ingestion, blood glucose concentrations were measured at 0, 20, 40, 60, 80, 100, and 120 min using a NIPRO Stat Strip XP2 (Nipro Corp., Osaka, Japan). The AUC was calculated using the trapezoid rule.

### 2.4. Quantitative Real-Time PCR Analysis

Total RNA was extracted from RNAlater-treated samples using QuickGene RNA tissue kit S II (Kurabo Industries Ltd., Osaka, Japan) according to the manufacturer’s protocol. cDNA was synthesized from total RNA using a High-Capacity cDNA Reverse Transcription Kit (Thermo Fisher Scientific K.K., Tokyo, Japan) according to the manufacturer’s protocol. Real-time quantitative PCR analysis was conducted using an AriaMx Real-Time PCR system (Agilent Technologies Japan, Ltd., Tokyo, Japan). The PCR solution was composed of SYBER Green supermix, each primer (10 µM), and a cDNA sample. The PCR cycling conditions were 95 °C for 1 min and 40 cycles of 95 °C for 15 s, followed by 60 °C for 1 min. The primer sequences for *Tnf-α*, *Il-6*, *Cox2 Inos,* and *GAPDH* were as follows: *Tnf-α*-F 5′-tccaggcggtgcctatgt-3′, *Tnf-α*-R 5′-gcccctgccacaagca-3′; *Il-6*-F 5′-ccacggccttccctacttc-3′, *Il-6*-R 5′-ttgggagtggtatcctctgtga-3′; *Cox2*-F 5′-tgcctcccactccagactaga-3′, *Cox2*-R 5′-cagctcagttgaacgcctttt-3′; *Inos*-F 5′- ggatcttcccaggcaacca -3′, *Inos*-R 5′-caatccacaactcgctccaa-3′; and *Gapdh*-F 5′-catggccttccgtgttccta-3′, *Gapdh*-R 5′-gcggcacgtcagatcca-3′.

### 2.5. Analysis of Metabolic Parameters in Feces and Cecal Contents

First, 0.1 g of fecal and cecum contents was mixed with 500 µL distilled water by vortex mixer for 5 min. Then, it was centrifuged at 1000 rpm for 5 min.

Short-chain fatty acids were analyzed using labeling reagents for long-chain and short-chain fatty acid analysis (YMC Co., Ltd., Kyoto, Japan) according to the manufacturer’s protocol. Briefly, 1 mL of extract above was mixed with 200 µL of reagent A and reagent B, then incubated at 60 °C for 20 min in water bath. Subsequently, it was mixed with 200 µL of reagent C, and incubated at 60 °C for 15 min. After mixing, 4 mL of reagent D and 5 mL of hexane was added and the mixture was centrifuged at 3000 rpm for 5 min. The supernatant was collected and evaporated with nitrogen gas. The residue was diluted with 200 µL of methanol and stored at −20 °C until HPLC analysis. After the labeling reagent was analyzed (ACQUITY UPLC H-Class PLUS system; Nihon Waters K.K., Tokyo, Japan) a reverse-phase column was used (ACQUITY UPLC HSS T3 1.8 µm, 2.1 mm × 100 mm; Nihon Waters K.K., Tokyo, Japan). The mobile phases were 0.1% formic acid water (solvent A) and 0.1% formic acid acetonitrile (solvent B). The linear gradient elution was performed as follows: time (*t* min) (*t*, A%): (0 min, 100%), (3 min, 70%), (7 min, 60%), (10 min, 60%), and (10.1 min, 100%), with a 0.3 mL/min flow rate, 5 μL injection volume, 210 nm wavelength, and 30 °C column temperature. The lactic acid, acetic acid, propionic acid, and butyric acid concentrations were calculated using the standard curve prepared using each standard reagent.

### 2.6. Microbiome Analysis

Using a feces collection kit (Techno Suruga Laboratory Co., Ltd., Shizuoka, Japan), DNA was extracted from mice fecal samples with the bead-beating method. The fecal samples in the solution, containing zirconia beads, were disrupted mechanically using a Precellys Evolution (Bertin Technologies SAS, FRA, Montigny-le-Bretonneux, France) at 8400 rpm, 2 × 60 s at room temperature. The homogenized sample was centrifuged at 2400× *g* for 1 min at room temperature. Then, 0.2 mL of the supernatant was collected. Thereafter, DNA was extracted using a GENE PREP STAR PI-480 (Kurabo Industries Ltd., Tokyo, Japan). The concentration of the extracted DNA was found using a NanoDrop Spectrophotometer ND-8000 (Thermo Fisher Scientific, Waltham, MA, USA). Next-generation sequencing analysis was performed using a MiSEq. (Illumina Inc., San Diego, CA, USA). The V3–V4 regions of bacterial 16S rRNA were amplified using the 341 F (5′-CCTACGGGAGGCAGCAG-3′) and 805R (5′-GACTACNVGGGTATCTAATCC-3′) [36] primers and the dual-index method [37].

Barcoded amplicons were paired-end sequenced on a 2 × 284 bp cycle using the MiSeq system with MiSeq Reagent Kit ver. 3 (600 Cycle) chemistry. Paired-end sequencing reads were merged using the fastq-join program with default settings (https://expressionanalysis.github.io/ea-utils/ (accessed on 7 May 2021). Only joined-reads that had a quality value score of ≥20 for more than 99% of the sequence were extracted using the FASTX-Toolkit (hannonlab.cshl.edu/fastx_toolkit/url (accessed on 7 May 2021). The chimeric sequences detected by QIIME1.8.0 were deleted for additional analysis. The filter-passed 16S rDNA reads were subjected to homology searching using Metagenome@KIN Ver 2.11 analysis software (World Fusion Co., Ltd., Tokyo, Japan) and RDP Classifier ver.2.11 with cut off for ≥0.8 [38]. Principal component analysis is often used as a tool in exploratory data analysis for variable dimensionality reduction; it is useful in reducing the high number of predictor variables to a few principal components. The effect of each test food on gut microbiota was examined using the principal component score. Furthermore, the presence of *Blautia* was calculated using 16S rRNA. The Kruskal–Wallis test was used to calculate *p* values.

### 2.7. Statistical Analysis

The results were expressed as mean ± standard deviation (SD). Statistical analyses between multiple groups were carried out using ANOVA. Statistically significant differences were estimated by Dunnett’s multiple comparison test. The Shirley–Williams multiple comparison test was used for Figure 4. The Student’s *t*-test was used for Figure 5. Significant difference was inferred for *p* < 0.05. Statistical analyses were conducted using software (Stat View-J ver. 5.0; SAS Institute Inc., Chicago, IL, USA).

## 3. Results

### 3.1. Growth Parameters and Relative Organ Weights

**Figure 1:**
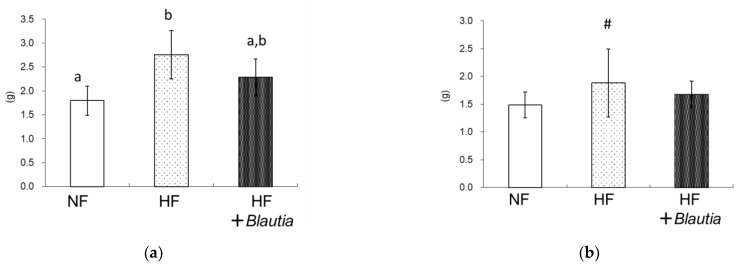

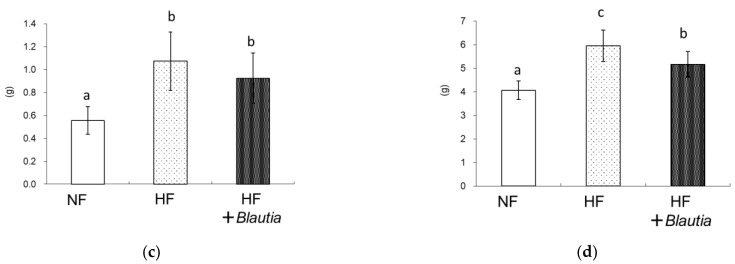
White adipose tissue (WAT) weight: (**a**) epididymal WAT, (**b**) mesenteric WAT, (**c**) retroperitoneal WAT, and (**d**) total WAT. Different letters (a, b, c) denote significance inferred for *p* < 0.05, # *p* = 0.08 vs. NF.

Table 2 presents growth parameters. The final body weight of the HF group was significantly higher (*p* < 0.05) than that of the NF group. However, the final body weight of the HF + *Blautia* group was different from that of the NF group. Total food intake, total water intake, and feces weight were not found to be different in the different groups. Table 3 shows data for organ weights. No significant difference was found in liver, spleen, kidney, cecum weights, or brown adipose tissue weights. However, the white adipose tissue (WAT) weights differed (Figure 1). Total WAT of the HF + *Blautia* group was significantly lower than those of the HF group (Figure 1d).

### 3.2. Serum Parameters

Table 4 presents the plasma parameters. Triglyceride, phospholipid, total cholesterol LDL, and HDL were not different among groups. Glutamic-oxaloacetic transaminase (GOT) and glutamic-oxaloacetic transaminase (GPT), which are indices of liver inflammation, were also unaffected.

### 3.3. Oral Glucose Tolerance Test

**Figure 2:**
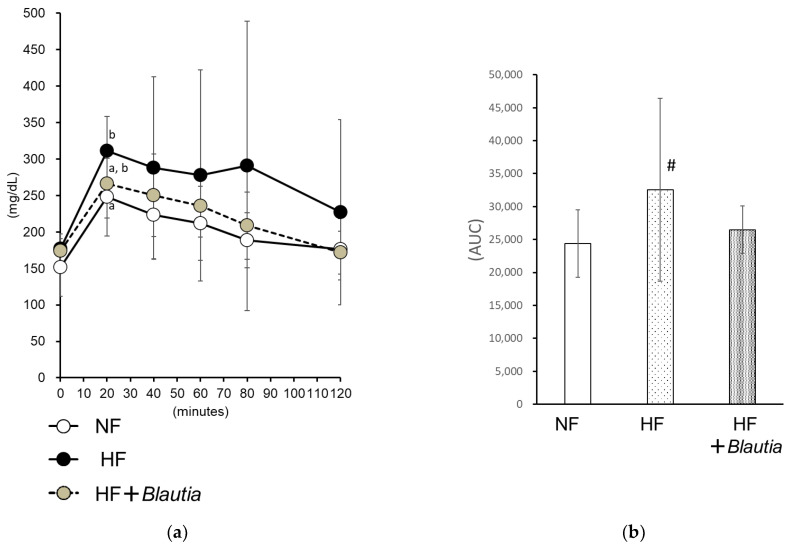
Glucose tolerance test: (**a**) blood glucose concentration of glucose tolerance test on C57BL/6J mice fed with experimental diets and (**b**) AUC level of blood glucose concentration on the glucose tolerance test. Different letters (a, b) denote significance inferred for *p* < 0.05, # *p* = 0.08 vs. NF.

Figure 2 presents glucose tolerance test results. After administration of glucose (4 mg/g of body weight), the glucose concentration of the HF-group mice was significantly (*p* < 0.05) higher than that of NF group at 20 min after administration. However, the concentration of the HF + *Blautia* group was not significantly higher (Figure 2a). Figure 2b shows the area under the curve (AUC) of the blood glucose concentration. The AUC levels of the HF group tended to be higher than those of NF group (*p* = 0.09). However, the level of the HF + *Blautia* group was not different from that of the NF group.

### 3.4. Short-Chain Fatty Acid Concentrations in Feces and Cecum

**Figure 3:**
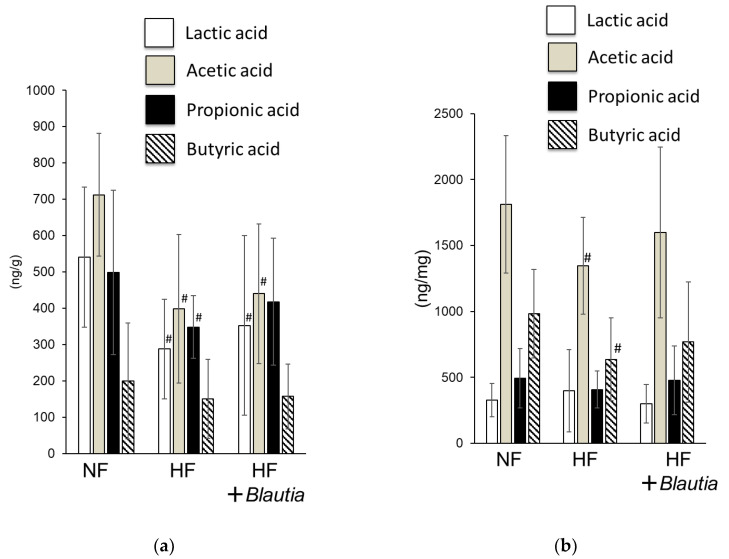
Short-chain fatty acid concentrations in the fecal and cecal contents: (**a**) fecal and (**b**) cecal contents. # *p* < 0.05 vs. NF.

Figure 3 presents the short-chain fatty acid concentrations in the feces and cecum. For feces, the lactic acid, acetic acid, and propionic acid concentrations of the HF group were significantly (*p* < 0.05) lower than those of the NF group (Figure 3a). However, the propionic acid concentration of the HF + *Blautia* group was not significantly different from that of the NF group. The acetic acid and butyric acid concentrations of the HF group were significantly (*p* < 0.05) lower than those of the NF group in cecal contents. By contrast, short-chain fatty acid concentrations of the HF + *Blautia* group were not different from those of the NF group (Figure 3b).

### 3.5. Serum Peptide YY Concentration

**Figure 4:**
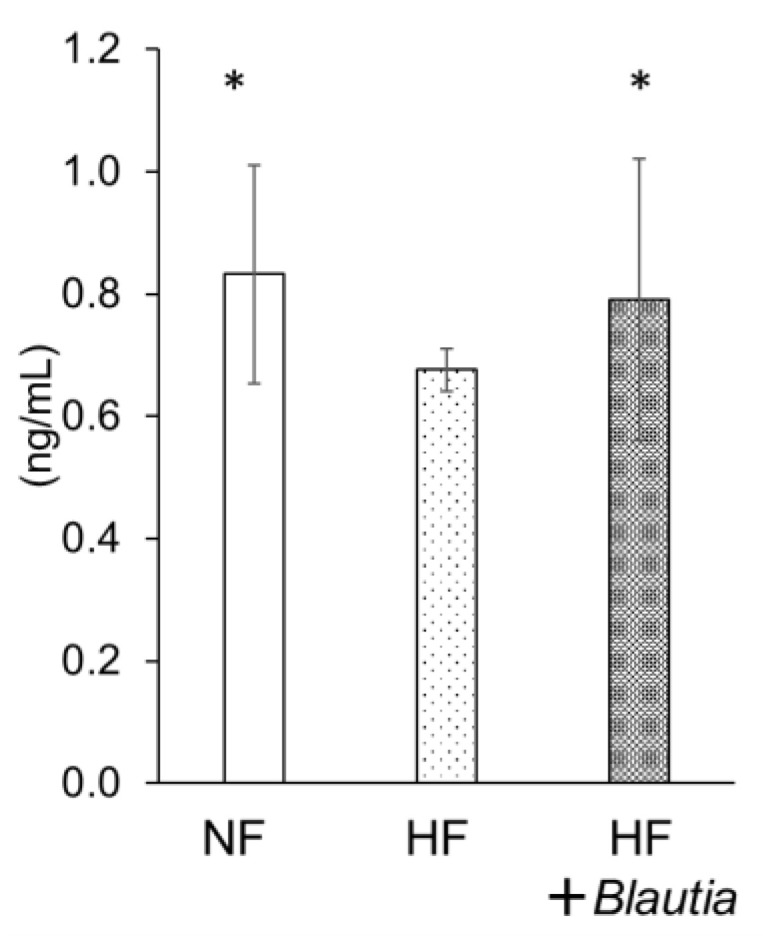
Serum peptide YY concentration * *p* < 0.05 vs. HF.

Figure 4 presents the serum peptide YY concentration. The peptide YY level of the HF + *Blautia* group was significantly (*p* < 0.05) higher than that of the HF group. The level was found to be equal to that of the NF group.

### 3.6. Gene Expression Related to Low-Grade Chronic Inflammation in the Liver

**Figure 5:**
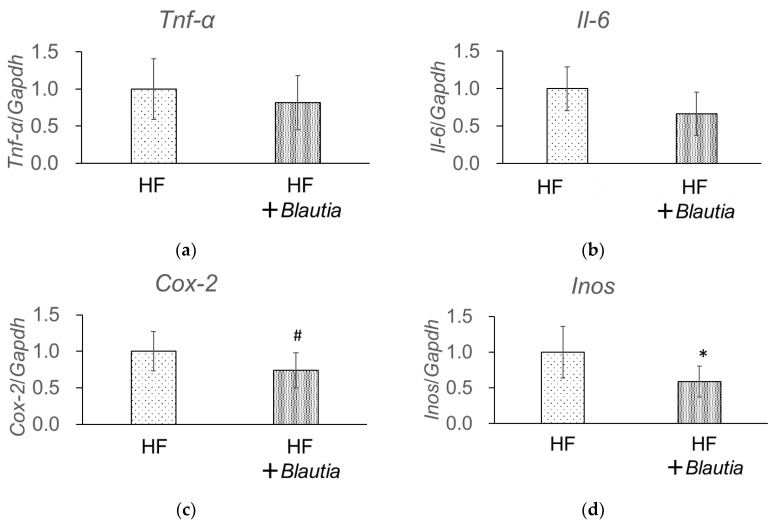
Levels of mRNA expression related to low-grade chronic inflammation glucose homeostasis in WAT were measured using real-time quantitative RT-PCR (Figure 3). Gene expression related to fatty acid synthesis and inflammation in the liver: (**a**) *Tnf-α*, (**b**) *Il-6*, (**c**) *Cox-2*, and (**d**) *Inos*. * *p* < 0.05 vs. HF, # *p* = 0.06 vs. HF.

Real-time quantitative RT-PCR (Figure 5) was used to measure the mRNA expressions related to low-grade chronic inflammation in the liver. Tumor necrosis factor α (*Tnf-α*), interleukin-6 (*Il-6*), cyclooxygenase-2 (*Cox-2*), and inducible nitric oxide synthase (*Inos*) are factors related to inflammation. *Cox-2* mRNA expression tended to be more suppressed in the HF + *Blautia* group than in the HF group (*p* = 0.06) (Figure 5c). In addition, *Inos* mRNA expression was significantly (*p* < 0.05) lower in the HF + *Blautia* group than in the HF group (Figure 5d).

### 3.7. Microbiota in Cecal Contents

**Figure 6:**
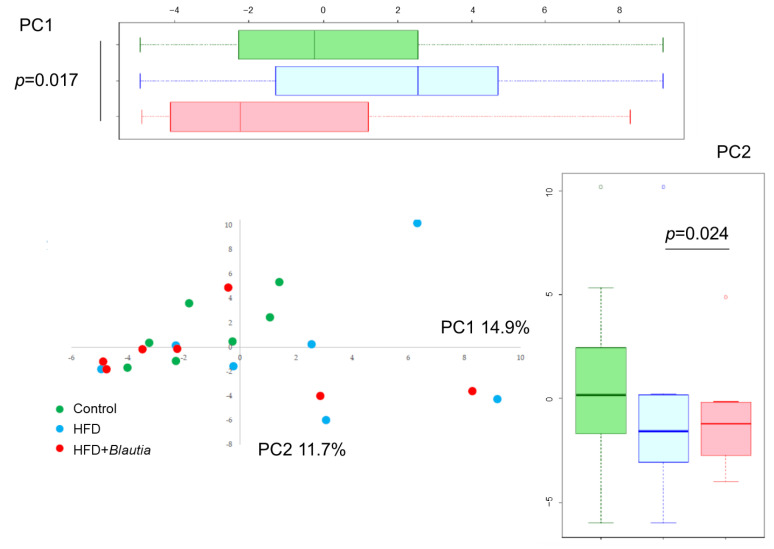
Microbiota in feces. PCA of the bacterial composition (genus level). Significance was inferred for *p* < 0.05.

**Figure 7:**
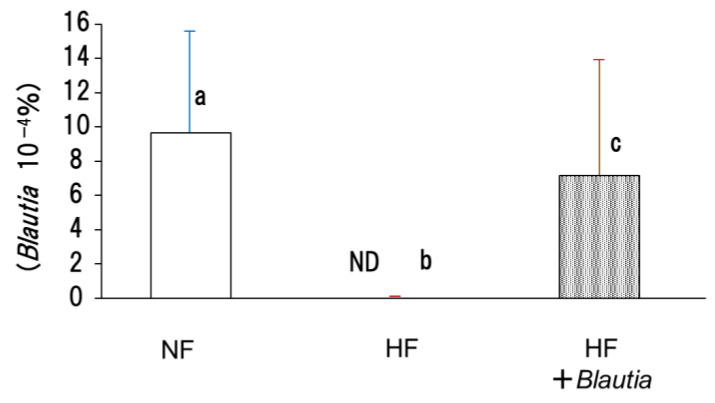
Relative *Blautia* abundances of feces. Different letters (a, b, c) denote significance inferred for *p* < 0.05.

PCA results showed primary components at the genus level (Figure 6). PC1 accounted for 14.9% of the variance for the genus level and PC2 accounted for 11.7%. Large distances among the NF, HF, and HF + *Blautia* groups were found (PC1). Furthermore, significant (*p* < 0.024) difference was found between HF and HF + *Blautia* groups (PC2). In addition, the HF diet group had significantly lower relative *Blautia* abundance than subjects with NF diets (Figure 7). The presence of *Blautia* in the HF group was below the detection limit. In contrast, the relative abundances of *Blautia* in the HF + *Blautia* group were significantly (*p* < 0.05) higher than those of the HF group.

## 4. Discussion

For this study, after *Blautia* was administered to mice with diet-induced obesity, the ameliorating effects of visceral fat accumulation and glucose homeostasis were investigated. The final body weights of the HF + *Blautia* group tended to be lower than those of the HF group. Also, the epididymal and total WAT weight of the HF + *Blautia* group were significantly lower than those of the HF group, suggesting that dietary *Blautia* bacteria have an inhibitory effect on obesity induced by a high-fat diet.

Furthermore, the HF + *Blautia* group showed similar behavior to that of the NF group for the glucose tolerance test. The AUC of the HF + *Blautia* group was not different from that of NF group. This result suggests an effect of improving the insulin resistance in the obesity condition.

In the HF + *Blautia* group, the mRNA expression level was related to inflammation of the liver and was more suppressed than in the HF group. The liver is a major organ in the regulation of glucose metabolism and lipid metabolism. In the obese condition, a low degree of chronic inflammation is induced in organs such as the liver, muscle, and adipose tissue [2,39]. White adipose tissue accumulation causes insulin resistance in organs related to glucose metabolism [40,41]. This inflammation is induced by inflammatory factors such as IL-6, TNF-α, COX-2, and iNOS. Actually, IL-6 and TNF-α are inflammatory cytokines secreted from macrophage cells or adipocytes. COX-2 promotes the production of prostaglandins from polyunsaturated fatty acids. iNOS is an enzyme that produces nitric oxide, which is an inflammatory mediator. Therefore, results suggest that the dietary *Blautia* suppressed a low degree of chronic inflammation induced by a high-fat diet, and led to the improvement in insulin resistance.

Dietary *Blautia* induced short-chain fatty acid production in the intestinal tract. This effect is regarded as related to the improvement in fat accumulation and insulin resistance. A high-fat diet downregulates intestinal microfloral diversity, especially the number of microbacteria producing short-chain fatty acids [42,43]. For this study, the amount of short-chain fatty acids in the contents of feces and cecum decreased or tended to decrease more in the HF group than in the NF group. However, the amounts of propionic acid in feces, acetic acid in the cecum, and butyric acid in HF + *Blautia* group were not different from those in the NF group. Therefore, dietary *Blautia* might be inferred as improving short-chain fatty acid production, which is otherwise suppressed by a high-fat diet. Short-chain fatty acids produced by intestinal microbiota stimulate short-chain fatty acid receptors such as GPR41 and GPR43, which regulate metabolism [30,31]. These metabolites from microbiota, which are also absorbed into the bloodstream from the large intestine, affect several organs throughout the body. The present study found that the serum concentration of peptide YY was significantly higher in the HF + *Blautia* group than in the HF group. Peptide YY is a gastrointestinal hormone that acts on the hypothalamus of the brain, exerting a suppressive effect on appetite [44]. Stimulation of short-chain fatty acids in the intestinal tract promotes the secretion of peptide YY. Therefore, short-chain fatty acid production by dietary *Blautia* is presumably related to metabolism-promoting effects.

In fact, *Blautia* characteristically promoted short-chain fatty acid production in an in vitro study [29]. The relative frequency of *Blautia hansenii* in feces was significantly higher in the HF + *Blautia* group than in the HF group, which suggests that administration of heat-sterilized cells of *Blautia hansenii* affects *Blautia hansenii* proliferation in the intestinal tract. Furthermore, the relative frequency of *Blautia* was significantly lower in the HF group than in the NF group, which is consistent with epidemiological investigations showing that people with higher visceral fat mass have fewer *Blautia* bacteria in their intestinal tract [24,25].

Consequently, correlation between visceral fat mass accumulation and *Blautia* suppression was shown.

Reportedly, short-chain fatty acid producing bacteria can proliferate if a person consumes indigestible food materials such as dietary fiber [26,42,44]. The bacterial constituents mainly comprise polysaccharides including peptide glucan [45]. In addition, yeast and fungal cells contain ceramide. Reportedly, the administration of ceramide increases the number of *Blautia* bacteria in intestinal microflora [46]. The possibility exists that the *Blautia* cell components promote *Blautia* proliferation. Dietary *Akkermansia muciniphila*, an intestinal bacterium, enhanced intestinal barrier functions and improved the energy metabolism in obese and diabetic mouse models [47]. The mechanism of this action has clarified as *Akkermansia muciniphila* cell wall proteins acting on the toll-like receptor (TLR2) of the host. In addition, administration of *Akkermansia muciniphila* reduces body weight and improves insulin sensitivity in obese patients [48]. In a three-month clinical trial, a diet supplemented with heat-sterilized *Akkermansia muciniphila* (1.0 × 10^11^ cfu/day) suppressed body weight, body fat weight, and total cholesterol level of serum, compared with a placebo group. This study also demonstrated safety in human trials. Therefore, dietary heat-sterilized *Blautia hansenii* might also proliferate and benefit the intestinal tract. However, the bioactivity and safety of oral administration of *Blautia hansenii* and safety have not been validated in clinical study. Further studies must clarify how dietary *Blautia hansenii* can promote their own growth in the human body.

## 5. Conclusions

In this study, the anti-obesity effects of *Blautia hansenii* were analyzed in a mouse model of high-fat-diet-induced obesity. Administration of *Blautia hansenii* suppressed the body weight gain and WAT accumulation, thereby improving insulin resistance. These effects were shown to be associated with the proliferation of *Blautia* in the intestinal tract and with promotion of the short-chain fatty acid production capacity. Administration of a high-fat diet reduced the number of *Blautia* in the intestinal tract. On the other hand, heat-sterilized *Blautia* cells proliferated in the intestinal tract. Based on findings of this study, food ingredients with the potential to promote *Blautia hansenii* proliferation, or the use of dietary *Blautia hansenii* as a food, are inferred as useful for preventing obesity-related diseases.

## Figures and Tables

**Table 1:** Compositions of diets used for animal experimentation (g/kg of diet).

Ingredients	NF	HF	HF + *Blautia*
Lard ^1^		130	130
Soybean oil ^2^	70	70	70
Corn starch ^3^	397.49	277.37	277.37
Casein ^3^	200	230.00	230.00
Dextrinized cornstarch ^3^	132.00	92.11	92.11
Sucrose ^4^	100	100	100
AIN-93 mineral mixture ^3^	35	35	35
AIN-93 vitamin mixture ^3^	10	10	10
L-cystine ^5^	3	3	3
Choline bitartrate ^5^	2.5	2.5	2.5
Cellulose ^3^	50	50	50
*Tert*-Butylhydroquinone ^5^	0.014	0.014	0.014
*Blautia* cells dry powder			0.04
Total	1000	1000	1000

^1^ Showa Chemical Ind. Co., Ltd., Tokyo, Japan. ^2^ Wako Pure Chemical Industries Ltd., Osaka, Japan. ^3^ CLEA Japan Inc., Tokyo, Japan.^4^ Kanto Chemical Co., Inc., Tokyo, Japan. ^5^ Sigma-Aldrich Corp., St. Louis, MO, USA.

**Table 2:** Growth parameters of C57BL/6J mice fed with the diets used for experimentation (g/100 g body weight).

	NF	HF	HF + *Blautia*
Initial body weight (g)	16.76 ± 1.40	16.78 ± 1.72	16.94 ± 1.42
Final body weight (g)	25.88 ± 1.25	28.13 ± 1.75 *	27.11 ± 1.75
Total food intake (g)	219.9 ± 9.6	204.0 ± 16.6	201.3 ± 15.3
Total water intake (g)	441.5 ± 51.1	406.8 ± 51.8	420.1 ± 45.7
Feces weight (g/2 days)	1.11 ± 0.18	0.99 ± 0.19	1.02 ± 0.15

* *p* < 0.05 vs. NF.

**Table 3:** Tissue weight of C57BL/6J mice fed with the diets used for experimentation (g/100 g body weight).

	NF	HF	HF + *Blautia*
Liver	3.81 ± 0.55	3.62 ± 0.49	3.77 ± 0.57
Spleen	0.28 ± 0.08	0.24 ± 0.07	0.25 ± 0.06
Kidney	1.28 ± 0.11	1.27 ± 0.18	1.27 ± 0.11
Cecum	1.73 ± 0.39	1.42 ± 0.30	1.51 ± 0.31
Brown adipose tissue	0.48 ± 0.09	0.53 ± 0.09	0.60 ± 0.11

**Table 4:** Plasma parameters and leptin concentrations of C57BL/6J mice fed with the diet used for experimentation.

	NF	HF	HF + *Blautia*
Triglyceride (mg/dL)	27.8 ± 14.5	25.6 ± 9.9	25.1 ± 12.7
Phospholipid (mg/dL)	258.1 ± 22.9	289.6 ± 22.9	285.0 ± 25.8
Total cholesterol (mg/dL)	123.4 ± 11.4	153.7 ± 10.5	148.7 ± 11.4
LDL cholesterol (mg/dL)	11.3 ± 1.7	18.0 ± 2.9	18.9 ± 3.2
HDL cholesterol (mg/dL)	105.7 ± 9.2	126.4 ± 6.8	122.9 ± 10.4
GOT (IU/L)	97.1 ± 45.0	66.6 ± 13.7	78.4 ± 34.8
GPT (IU/L)	19.0 ± 4.1	15.6 ± 2.1	19.0 ± 6.1

## Data Availability

The datasets generated and analyzed in the current study are available from the corresponding author on reasonable request.
